# The association between white matter changes and development of malignant middle cerebral artery infarction: A case–control study

**DOI:** 10.1097/MD.0000000000025751

**Published:** 2021-04-30

**Authors:** Meng-Ni Wu, Pen-Tzu Fang, Chih-Hsien Hung, Chung-Yao Hsu, Ping-Song Chou, Yuan-Han Yang

**Affiliations:** a Department of Neurology; b Department of Radiation Oncology, Kaohsiung Medical University Hospital; c Department of Neurology, Kaohsiung Municipal Ta-Tung Hospital, Kaohsiung, Taiwan).

**Keywords:** brain edema, ischemic stroke, leukoaraiosis, middle cerebral artery, white matter changes

## Abstract

Disrupted blood–brain barrier (BBB) in patients with ischemic stroke plays a critical role in malignant middle cerebral artery infarction (MMI) development.

Cerebral white matter changes (WMC), particularly in the deep subcortical area or in severe one, may be also underlain by disrupted BBB. It is unclear whether the presence of WMC with potential premorbid disruption of BBB makes patients susceptible to MMI. Therefore, this study aimed to clarify any putative relationship between the MMI and WMC in terms of their severity and locations.

In this case–control study, patients with infarction in the middle cerebral artery territory were retrospectively reviewed. Brain magnetic resonance images were analyzed according to Fazekas scale, and identified WMC were divided into periventricular WMC (PV-WMC) and deep subcortical WMC (deep-WMC). Patients were scored as having WMC, PV-WMC, deep-WMC, severe PV-WMC, and severe deep-WMC according to the severity and locations. Patients were defined as having MMI if either a progressive conscious disturbance or signs of uncal herniation was recorded in combination with a midline shift >5 mm identified on the follow-up computed tomography.

Among 297 patients admitted between July 2009 and February 2015, 92 patients were eligible for final analysis. Compared to patients without MMI, patients with MMI had a higher score of National Institutes of Health Stroke Scale, a larger infarct volume, and an increasingly greater proportion of severe PV-WMC, deep-WMC, and severe deep-WMC, respectively. After adjustment for sex, age, infarct volume, and history of hypertension, severe deep-WMC (odds ratio [OR] = 6.362, 95% confidence interval [CI] = 1.444–28.023, *P *= .0144) and severe PV-WMC (odds ratio = 5.608, 95% confidence interval = 1.107–28.399, *P* = .0372) were significantly associated with MMI development.

MMI and WMC are significantly associated such that MMI development is more likely when PV-WMC or deep-WMC is more severe. We hypothesize that Fazekas scale-defined severe deep-WMC and PV-WMC may be considered as clinically approachable predictors of MMI development. These findings support that the WMC with potential premorbid disrupted BBB may make patients susceptible to MMI, and further prospective study should be conducted to clarify this hypothesis.

## 1 Introduction

Approximately 10% of patients with infarction in the territory of the middle cerebral artery (MCA) develop a progressive conscious disturbance or signs of herniation with a shift of midline structure, which was defined as a malignant middle cerebral artery infarction (MMI).^[1]^ Patients with MMI may progress to demanding disability and/or a vegetative state; in fact, the MMI mortality rate is 80% if untreated.^[1,2]^ Because early decompressive hemicraniectomy within 48 hours after onset reduces mortality and improves the functional outcome,^[3,4]^ early identification of patients at risk and prediction of malignant course are essential.

It has been reported that young age, being female, a history of hypertension, and congestive heart failure, decreased collateral circulation, severity of stroke, and infarct volume are associated with development of MMI.^[5–11]^ Researches on predictors of MMI development seem to focus on demographic characteristics and imaging findings. Conversely, only a limited number of studies have investigated predictors of MMI development in the pathophysiological terms. In the early stage of infarction, ischemia initiates cytotoxic brain edema which drives further ionic edema with transcellular and transcapillary influx of water and ions. Afterward, disruption of blood–brain barrier (BBB) accounts for continuous extravasation of fluid and macromolecules in the stage of vasogenic brain edema, which plays a critical role in MMI development.^[12–14]^ However, it is still unclear whether premorbid disrupted BBB integrity makes patients with MCA infarction vulnerable to vasogenic edema.

Cerebral white matter changes (WMC) are frequently observed in routine brain computed tomography (CT) and magnetic resonance imaging (MRI) in elderly people.^[15–17]^ The presence of WMC is associated with different stages of ischemia, with arteriosclerotic changes and dysfunction of BBB in a potential exposure-response relationship.^[18–24]^ Furthermore, WMC in the deep subcortical area with confluence were reported to be related to extensive ischemic or other microangiopathic changes, including disrupted BBB, increasing cerebrovascular resistance, and impaired cerebral autoregulation.^[23–25]^ Although dysfunction of BBB may underlie both MMI development and some types of WMC, to the best of our knowledge, no study had discussed the potential relationship between WMC and MMI except for one study reporting the relationship between WMC and compression of ventricles regardless of clinical deterioration.^[26]^

On the basis of the hypothesis that there is an association between the MMI development and WMC, this study was designed to clarify any putative relationship between the MMI and WMC in terms of their severity and locations.

## 2 Methods

In this case–control study, information of patients with a confirmed infarction of MCA territory who were admitted to the Neuro-intensive Care Unit of Kaohsiung Medical University Hospital between July 2009 and February 2015 were retrospectively analyzed. The informed consent is not applicable because of the retrospective design of this study. The hospital's Institutional Review Board approved this study (KMUH-IRB-E- 20150032).

### 2.1 Patients

Upon admission, all patients underwent complete physical and neurological examinations, the assessment of National Institutes of Health Stroke Scale (NIHSS), laboratory surveys, and brain CT scans. During hospitalization, patients underwent brain MRI, including diffusion-weighted imaging (DWI), and T2-weighted-fluid-attenuated inversion recovery (FLAIR) imaging. If the patient's neurological condition deteriorated, a follow-up brain CT scan was performed. Demographic characteristics, baseline laboratory results, and medical histories were collected from patients’ medical records.

Congestive heart failure was determined based on chart review. Hypertension was diagnosed if there was a recorded history of hypertension or usage of antihypertensive medications. Diabetes was diagnosed if there was a recorded history of diabetes, or a fasting sugar ≥126 mg/dL combined with a glycated hemoglobin >6.5% identified after admission. Atrial fibrillation was diagnosed if there was a recorded history of atrial fibrillation or if an atrial fibrillation was identified in any 12-lead electrocardiogram or 24-hour Holter electrocardiogram results after admission.

A follow-up brain CT was arranged if patients had either a progressive conscious disturbance (decreased level of consciousness ≥1 on item 1a of NIHSS or decline ≥4 on total NIHSS) or clinical signs of uncal herniation. Patients were considered to have development of MMI if the follow-up CT revealed a midline shift >5 mm at the level of septum pellucidum, which indicate the decompressive hemicraniectomy.^[9,27]^

Patients who met the following criteria were included: precisely established onset of symptoms; brain MRI scan performed within 48 hours after onset; infarction involving MCA territory confirmed by DWI; infarct volume ≥82 mL calculated from the DWI data.^[28,29]^

**Figure 1:**
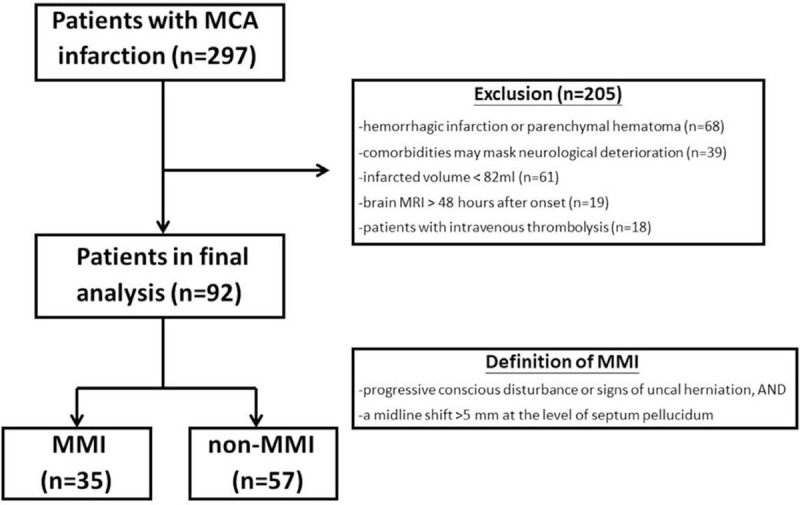
Algorithm for identifying patients fulfilled criteria and divided into MMI and non-MMI groups. MCA = middle cerebral artery, MMI = malignant middle cerebral artery infarction.

Patients were excluded if they had any of following conditions: receiving intravenous thrombolysis or mechanical thrombectomy; any hemorrhagic transformation (either hemorrhagic infarction or parenchymal hematoma) identified on MRI; involvement of other vascular territory; evidence of midline shift already identified on MRI; preexisting encephalomalacia or intracranial mass affecting the course of brain edema; other comorbidities potentially masking neurological deterioration, including seizure, sepsis, shock, severe hyponatremia (serum sodium <125 mg/dL) or hypernatremia (serum sodium >155 mg/dL), or severe hypoglycemia (serum glucose <60 mg/dL) within 5 days after infarction. Figure 1 demonstrates the algorithm for identifying patients fulfilled criteria and how to divide them into MMI and non-MMI groups

### 2.2 Magnetic resonance imaging

All MRI examinations were conducted using a 3.0-T MRI (GE Medical Systems). The DWI sequence was composed of 25 slices (slice thickness 6 mm) with a 1-mm interslice gap. Through trace analyses of DWI images (b = 1000 s/mm^2^), hyperintense signals were manually selected by one of the authors (PTF), and the infarct volume was calculated as the summation of the hyperintense area in each transection multiplied by slice thickness.^[30]^ The intrarater reliability was represented with an intraclass correlation coefficient (ICC) of 0.862.

### 2.3 Grading of WMC on MRI

On the basis of Fazekas scale,^[17]^ WMC were visually scored using the FLAIR images in the periventricular and deep subcortical areas, respectively, by one of the authors (MNW), who was blinded to the patient's MMI status. The intrarater reliability was represented with an ICCs of 0.972 and 0.964 in the periventricular and deep subcortical areas, respectively.

**Figure 2:**
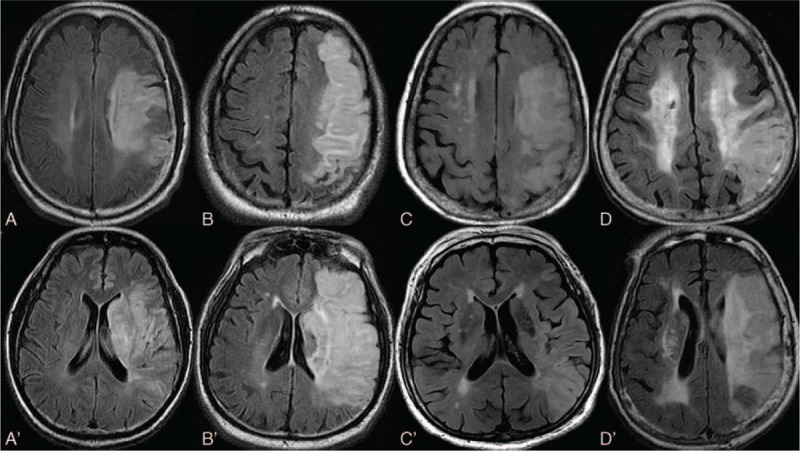
Demonstration of periventricular (PV) white matter changes (WMC) with different locations and severities. White matter changes in deep subcortical area (deep-WMC) were demonstrated in A (grade 0), B (grade 1), C (grade 2), and D (grade 3), whereas white matter changes in periventricular area (PV-WMC) were demonstrated in A’ (grade 0), B’ (grade 1), C’ (grade 2), D’ (grade 3).

The severity of WMC in the periventricular area (PV-WMC) was graded as follows: 0, no lesion; 1, cap or pencil-thin lining; 2, small-halo; 3, irregular periventricular WMC extending into deep white matter. The severity of WMC in the deep subcortical area (deep-WMC) was graded as follows: 0, no lesion; 1, punctate lesion; 2, beginning confluence of lesion; 3, large confluence of lesion.^[17]^Figure 2 demonstrates different grade of WMC in periventricular and deep subcortical area. Patients were defined as having WMC if there were any identifiable WMC regardless of severity or location (either grade of PV-WMC ≥1 or grade of deep-WMC ≥1). Patients were defined to have PV-WMC and deep-WMC if graded with any non-zero grade on the region-specific Fazekas scale (PV- or deep-WMC) regardless of severity.

Patients were defined as having severe PV-WMC if WMC with PV-WMC grade 2 or grade 3 were identified in the periventricular area. Patients were defined as having severe deep-WMC if WMC with deep-WMC grade 2 or grade 3 were identified in the deep subcortical area.

### 2.4 Statistical analysis

Statistical analyses were carried out using JMP Software version 9 (SAS Institute, Cary, NC). The categorical data were presented as values with the proportion of patients and analyzed by *χ*^2^ tests. The continuous data were presented as mean ± standard deviation (SD) and analyzed by Student *t* test. Variables with a *P* value <.1 in univariate analysis and clinically meaningful variables were entered into further multivariate logistic regression analyses. Logistic regression analyses were conducted with the dependent variable set as MMI development, and the independent variable set as severe PV-WMC, deep-WMC, and severe deep-WMC, respectively, in each model after adjustment for sex, age, infarct volume, and history of hypertension. Although both NIHSS and infarct volume represented the severity of stroke, only infarct volume was chosen into logistic regression under the consideration of NIHSS potentially varied in dominant and nondominant hemisphere. A *P* value <.05 was considered statistically significant.

## 3 Results

Among the 297 patients with infarction involving MCA territory, 205 patients were excluded for the following criteria: 68 patients with hemorrhagic infarction or parenchymal hematoma, 39 with other comorbidities potentially masking neurological deterioration within 5 days, 61 with infarct volume <82 mL, 19 undergoing brain MRI examination >48 hours after onset, and 18 receiving intravenous thrombolytic therapy. The remaining 92 patients were eligible for further analysis.

Table 1 shows demographic data, infarct volumes, NIHSS scores on admission, and types of WMC between the MMI and non-MMI groups. Thirty-five patients (38%) developed MMI, but the remaining 57 patients (62%) did not. Patients in the MMI group had significantly higher NIHSS scores on admission (18.7 ± 5.5 vs 16.4 ± 4.9, *P* = .0380) and larger infarct volumes (245.0 ± 74.7 mL vs 147.4 ± 53.6 mL, *P* < .0001) than those in the non-MMI group. There were 44 women (47.8%) enrolled in this study. Women had a higher prevalence of hypertension (84.1% vs 62.5%, *P* = .0200) and were older (76.4 ± 10.0 vs 66.4 ± 12.3 years, *P* < .0001) than men in the study. The interval between onset of stroke and MRI scanning was not significantly different between groups (1.60 ± 0.59 days vs 1.51 ± 0.51 days, *P* = .4978).

**Table 1:** Demographic characteristics and white matter changes of patients in malignant middle cerebral artery infarction and nonmalignant middle cerebral artery infarction group.

	Total (n = 92)	Non-MMI (n = 57)	MMI (n = 35)	*P*
Age, y, mean (±SD)	71.2 (12.3)	70.9 (12.4)	71.7 (12.2)	.7794
NIHSS, score, mean (±SD)	17.3 (5.2)	16.4 (4.9)	18.7 (5.5)	.0380^∗^
Infarct volume, mL, mean (±SD)	184.5 (72.2)	147.4 (53.6)	245.0 (74.7)	<.0001^∗^
Sex (male), n (%)	48 (52.2)	32 (56.1)	16 (45.7)	.3311
Hypertension, n (%)	67 (72.8)	38 (66.7)	29 (82.9)	.0901
Diabetes, n (%)	44 (48.4)	26 (45.6)	18 (52.9)	.4986
Congestive heart failure, n (%)	49 (53.3)	28 (49.1)	21 (60.0)	.3100
Atrial fibrillation, n (%)	41 (44.6)	23 (40.4)	18 (51.4)	.2993
Presence of any WMC, n (%)	60 (65.2)	33 (57.9)	27 (77.1)	.0598
PV-WMC				
Any PV-WMC, n (%)	56 (60.9)	31 (54.4)	25 (71.4)	.1039
Severe PV-WMC, n (%)	40 (43.5)	18 (31.6)	22 (62.9)	.0033^∗^
Deep-WMC				
Any deep-WMC, n (%)	51 (55.4)	26 (45.6)	25 (71.4)	.0156^∗^
Severe deep-WNC, n (%)	32 (34.8)	12 (21.1)	20 (57.1)	.0004^∗^

Among the 92 patients, 60 (65.2%) demonstrated WMC, 56 (60.9%) had PV-WMC, 40 (43.5%) had severe PV-WMC, 51 (55.4%) had deep-WMC, and 32 (34.8%) had severe deep-WMC. The 35-patient subgroup with MMI was significantly more likely to have severe PV-WMC (62.9% vs 31.6%, *P* = .0033), deep-WMC (71.4% vs 45.6%, *P* = .0156), and severe deep-WMC (57.1% vs 21.1%, *P* = .0004), respectively, than the patients who did not develop MMI. The proportion of MMI and non-MMI subgroups demonstrating any WMC or PV-WMC was not significantly different between groups. The patient group with PV-WMC had a significantly higher proportion of comorbid deep-WMC (83.9% vs 11.1%, *P* < .0001) than did the group without PV-WMC. The patient group with severe PV-WMC had a significant higher proportion of comorbid severe deep-WMC (75.0% vs 3.9%, *P* < .0001) than did the group without severe PV-WMC.

Patients with each kind of WMC, including the presence of any WMC, PV-WMC, severe PV-WMC, deep-WMC, and severe deep-WMC, had a significantly greater incidence of hypertension and congestive heart failure, were more likely to be women, and were significantly older (summarized in Supplemental Table S1–S5).

The logistic regression analysis results are provided in Tables 2 and 3. After adjustment for sex, age, infarct volume and history of hypertension, the severe deep-WMC (odds ratio [OR] = 6.362, 95% confidence interval [CI] = 1.444–28.023, *P* = .0144) and severe PV-WMC (OR = 5.608, 95% CI = 1.107–28.399, *P* = .0372) were significantly associated with the MMI development. There was no significant association between MMI and only deep-WMC (OR = 1.991, 95% CI = 0.489–8.109, *P* = .3363) after adjusting for the same confounding variables described above.

**Table 2:** Logistic regression analysis of development of malignant middle cerebral artery infarction to the severe deep white matter changes.

	OR	MMI (crude)		OR	MMI (adjusted)	
		95% CI	*P*		95% CI	*P*
Sex (male)	0.658	0.282–1.533	.3321	0.265	0.053–1.315	.1043
Age, y	1.005	0.971–1.040	.7765	0.945	0.889–1.005	.0719
Infarct volume, mL	1.024	1.014–1.033	<.0001^∗^	1.029	1.017–1.041	<.0001^∗^
Hypertension	2.417	0.856–6.819	.0955	1.313	0.274–6.294	.7335
Severe Deep-WMC	5.000	1.985–12.596	.0006^∗^	6.362	1.444–28.023	.0144^∗^

**Table 3:** Logistic regression analysis of development of malignant middle cerebral artery infarction to the severe periventricular white matter changes.

	OR	95% CI	*P*
Sex (male)	0.218	0.044–1.082	.0624
Age, y	0.924	0.859–0.994	.0337^∗^
Infarct volume, mL	1.028	1.016–1.040	<.0001^∗^
Hypertension	1.648	0.349–7.777	.5282
Severe PV-WMC	5.608	1.107–28.399	.0372^∗^

## 4 Discussion

The present study revealed a significant association between MMI development and some types of WMC; it also suggests that the severity of WMC correlates positively with this association. The severe WMC, including severe deep-WMC and severe PV-WMC, were significantly and positively related to MMI development. Conversely, no significant relationship was recorded between MMI development and having only WMC, PV-WMC, or deep-WMC.

The variable pathophysiology underlying WMC with differing severity and locations may explain the varying relationship between MMI and different WMC. PV-WMC with periventricular caps (PV-WMC grade 1) are related to a loosened arrangement of nerve fibers, and PV-WMC with small halos (PV-WMC grade 2) are related to a disrupted ependymal lining.^[23,24]^ Conversely, PV-WMC that extends to the deep subcortical area (PV-WMC grade 3) are significantly and independently determined by vascular origin.^[18,23]^ The deep-WMC with punctate lesions (deep-WMC grade 1) are related to reduced myelin, whereas deep-WMC with any type of confluence (deep-WMC grade 2 or 3) are related to extensive ischemic or other microangiopathic changes, including disrupted BBB, increasing cerebrovascular resistance, and impaired cerebral autoregulation.^[18–24]^ In this study, patients with severe deep-WMC, defined as deep-WMC grade 2 or 3, and some patients with severe PV-WMC, including those with PV-WMC grade 3, may potentially have premorbid disrupted BBB with excessive permeability, and may be prone to be vulnerable to vasogenic edema.

In this study, PV-WMC with small halos (PV-WMC grade 2) are also defined as part of severe PV-WMC, although it did not bear potential microangiopathic changes. However, approximately 75% of patients with severe PV-WMC concurrently had severe deep-WMC, which may partially explain why severe PV-WMC was reported to be associated with MMI development.

Muscari et al reported that white matter lesions, defined as either WMC restricted in the periventricular area or extending to cortex, may have a protective effect on compression of lateral ventricles, regardless of neurological deterioration.^[26,31]^ In that study, no further analysis was conducted based on severity and location of WMC even though variable pathophysiologic mechanism underlies WMC with different severities and locations, which may misinterpret the impact of WMC on brain edema. Furthermore, MMI, treated as a status with secondary brain insult, is usually defined as neurological deterioration in addition to evidence of brain edema with not only compression of ventricles but also midline shift. Therefore, we defined MMI as a progressive conscious disturbance or signs of uncal herniation with evidence of midline shift >5 mm and analyzed the relationship between MMI and WMC regarding the severity and location.

The decreased collateral circulation scale, reduced perfusion and increased BBB permeability on perfusion CT scan, and severe ischemia in positron emission tomography scan have been reported to predict MMI.^[30,32–34]^ However, these methods are relatively facility-dependent and time-consuming, which make them less clinically approachable. Conversely, the Fazekas scale is well-established, convenient, and reliable for scoring WMC in a routine, noncontrast brain CT or MRI.^[17]^ Here, we used Fazekas scale to demonstrate the relationship between severe WMC and MMI development. The data support that using the Fazekas scale defined severe WMC may be a clinically approachable method to predict MMI development. Further studies must be conducted to verify our findings in a larger patient cohort.

The area of infarction, either measured by the involved percentage (>50%–66%) of MCA territory, the Alberta Stroke Program Early CT Score (ASPECTS, ≤7), or a defined volume of infarction >145 mL, appears related to MMI development.^[5,6,9,11,28]^ Similarly, this study revealed that the infarct volume is a strong predictor of MMI development. The prevalence of MMI had been reported as 12.5% in supratentorial infarction, whereas 38% of the patients developed MMI in this study.^[35]^ Patients were enrolled in this study only if the infarct volume was >82 mL. These patients already have a high likelihood of developing MMI,^[29]^ which may explain its relatively high prevalence in this study. It has also been reported that the chance of developing MMI increases 1.16 times for every point increase on the NIHSS; notably, the NIHSS score in patients with MMI is usually >18.^[10,29,36]^ This study revealed that the mean NIHSS score in our MMI group (with a mean of 18.7 ± 5.5) is significantly higher than that in the non-MMI group, which supports the findings of the previous studies cited above.

There were some limitations in this study. First, the effects of severity and duration of hypertension, diabetes, and congestive heart failure on MMI development could not be clarified because of the retrospective design. Second, we could not control for having different clinicians to determine the NIHSS score and the interval between onset of stroke and evaluation of NIHSS scores. However, all clinicians were neurologists, and all NIHSS scores were evaluated on the admission day. Third, the precise interval between onset of stroke and brain MRI scan varied, which may affect the comparison of infarct volume between groups. However, only MRI scans conducted within 48 hours after the stroke symptoms onset were included in this study, and no significant difference of interval (measured in day) between onset and performance of MRI scan was identified between groups. Fourth, only patients at risk for developing MMI (infarct volume >82 mL) were included in this study,^[29]^ which may affect generalizability. Fifth, because of the retrospective design, we cannot precisely clarify the status of reperfusion by means of CT- or conventional angiography. Therefore, we excluded patients who received intravenous thrombolysis or mechanical thrombectomy to reduce impact of reperfusion, which may also affect generalizability. Sixth, the Fazekas scale can only be evaluated on the hemisphere contralateral to infarction, which may underestimate the severity of WMC in this study. However, the distribution of WMC is usually bilaterally symmetric in patients with severe WMC.^[37]^

## 5 Conclusions

In conclusion, the present study revealed a significant association between MMI development and WMC that varies with the severity of WMC. Only the presence of severe deep-WMC and severe PV-WMC was significantly associated with MMI development. We speculated that Fazekas scale-defined severe deep-WMC and severe PV-WMC may be considered as reliable and clinically approachable predictors for the MMI development. Furthermore, these findings may support that the premorbid disrupted BBB integrity, surrogated by severe WMC, may be vulnerable to MMI, and further prospective study should be conducted to clarify this hypothesis.

## Acknowledgments

The authors are grateful to Mr. Yu-Tsang Wang, a statistician in department of medical research of Kaohsiung Medical University Hospital, for statistical consultation.

## Author contributions

**Conceptualization:** Meng-Ni Wu, Chung-Yao Hsu, Yuan-Han Yang.

**Data curation:** Meng-Ni Wu, Ping-Song Chou.

**Formal analysis:** Meng-Ni Wu, Pen-Tzu Fang, Chih-Hsien Hung, Chung-Yao Hsu, Yuan-Han Yang.

**Funding acquisition:** Yuan-Han Yang.

**Methodology:** Meng-Ni Wu, Pen-Tzu Fang, Chih-Hsien Hung, Chung-Yao Hsu, Yuan-Han Yang.

**Resources:** Meng-Ni Wu.

**Software:** Meng-Ni Wu, Pen-Tzu Fang.

**Supervision:** Pen-Tzu Fang, Chih-Hsien Hung, Yuan-Han Yang.

**Validation:** Meng-Ni Wu, Pen-Tzu Fang, Chih-Hsien Hung, Ping-Song Chou, Yuan-Han Yang.

**Visualization:** Meng-Ni Wu, Pen-Tzu Fang, Chih-Hsien Hung, Ping-Song Chou, Yuan-Han Yang.

**Writing – original draft:** Meng-Ni Wu, Pen-Tzu Fang, Yuan-Han Yang.

**Writing – review & editing:** Meng-Ni Wu, Chih-Hsien Hung, Chung-Yao Hsu, Yuan-Han Yang.

## Supplementary Material

Supplemental Digital Content

## Supplementary Material

Supplemental Digital Content

## Supplementary Material

Supplemental Digital Content

## Supplementary Material

Supplemental Digital Content

## Supplementary Material

Supplemental Digital Content
