# Supplementary material for: The association between white matter changes and development of malignant middle cerebral artery infarction: A case–control study
Source: Medicine (Baltimore). 2021 Apr 30;100(17):e25751. doi: 10.1097/MD.0000000000025751 (PMC8084049; doi:10.1097/MD.0000000000025751)
Supplement: Supplemental Digital Content [file medi-100-e25751-s001.doc]

Table S1. Demographic data of patients with and without any WMC

|  | Non-WMC  (n=32) | WMC  (n=60) | *p*-value |
| --- | --- | --- | --- |
| Sex (male), n (%) | 23 (71.9) | 25 (41.7) | 0.0057* |
| Age, years, mean (±SD) | 64.6 (10.9) | 74.7(11.6) | 0.0001* |
| A-fib, n (%) | 11 (34.4) | 30(50.0) | 0.1510 |
| Hypertension, n (%) | 17 (53.1) | 50(83.3) | 0.0019* |
| Diabetes, n (%) | 14 (43.8) | 30 (50.0) | 0.5676 |
| Congestive heart failure, n (%) | 11 (34.4) | 38 (63.3) | 0.0080* |

* *p*<0.05

WMC, white matter changes; A-fib, atrial fibrillation

Patients with any WMC had a significantly greater incidence of hypertension and congestive heart failure, were more likely to be women, and were significantly older in comparison to those without any WMC
