# Supplementary material for: The association between white matter changes and development of malignant middle cerebral artery infarction: A case–control study
Source: Medicine (Baltimore). 2021 Apr 30;100(17):e25751. doi: 10.1097/MD.0000000000025751 (PMC8084049; doi:10.1097/MD.0000000000025751)
Supplement: Supplemental Digital Content [file medi-100-e25751-s002.doc]

Table S2. Demographic data of patients with and without PV-WMC

|  | Non-PV-WMC  (n=36) | PV-WMC  (n=56) | *p*-value |
| --- | --- | --- | --- |
| Sex (male), n (%) | 25 (69.4) | 23 (41.1) | 0.0078* |
| Age, years, mean (±SD) | 63.8 (11.0) | 76.0 (10.6) | <0.0001* |
| A-fib, n (%) | 12 (33.3) | 29 (51.8) | 0.0822 |
| Hypertension, n (%) | 20 (55.6) | 47 (83.9) | 0.0028* |
| Diabetes, n (%) | 15 (41.7) | 29 (51.8) | 0.3430 |
| Congestive heart failure, n (%) | 13 (36.1) | 36 (64.3) | 0.0082* |

* *p*<0.05

WMC, white matter changes; A-fib, atrial fibrillation

Patients with PV-WMC had a significantly greater incidence of hypertension and congestive heart failure, were more likely to be women, and were significantly older in comparison to those without PV-WMC.
