# Supplementary material for: The association between white matter changes and development of malignant middle cerebral artery infarction: A case–control study
Source: Medicine (Baltimore). 2021 Apr 30;100(17):e25751. doi: 10.1097/MD.0000000000025751 (PMC8084049; doi:10.1097/MD.0000000000025751)
Supplement: Supplemental Digital Content [file medi-100-e25751-s003.doc]

Table S3. Demographic data of patients with and without severe PV-WMC

|  | Non-Severe  PV-WMC  (n=52) | Severe  PV-WMC  (n=40) | *p*-value |
| --- | --- | --- | --- |
| Sex (male), n (%) | 34 (65.4) | 14 (35.0) | 0.0038* |
| Age, years, mean (±SD) | 65.8 (11.9) | 78.2 (8.8) | <0.0001* |
| A-fib, n (%) | 17 (32.7) | 24 (60.0) | 0.0090* |
| Hypertension, n (%) | 31 (59.6) | 36 (90.0) | 0.0012* |
| Diabetes, n (%) | 26 (50.0) | 18 (45.0) | 0.6341 |
| Congestive heart failure, n (%) | 21 (40.4) | 28 (70.0) | 0.0048* |

* *p*<0.05

WMC, white matter changes; A-fib, atrial fibrillation

Table S3 Patients with severe PV-WMC had a significantly greater incidence of hypertension and congestive heart failure, were more likely to be women, and were significantly older in comparison to those without severe PV-WMC.
