# Supplementary material for: The association between white matter changes and development of malignant middle cerebral artery infarction: A case–control study
Source: Medicine (Baltimore). 2021 Apr 30;100(17):e25751. doi: 10.1097/MD.0000000000025751 (PMC8084049; doi:10.1097/MD.0000000000025751)
Supplement: Supplemental Digital Content [file medi-100-e25751-s004.doc]

Table S4. Demographic data of patients with and without Deep-WMC

|  | Non-deep-WMC  (n=41) | deep-WMC  (n=51) | *p*-value |
| --- | --- | --- | --- |
| Sex (male), n (%) | 29 (70.7) | 19 (37.3) | 0.0014* |
| Age, years, mean (±SD) | 66.3 (11.5) | 75.1 (11.6) | 0.0005* |
| A-fib, n (%) | 14 (34.2) | 27 (52.9) | 0.0714 |
| Hypertension, n (%) | 21 (51.2) | 46 (90.2) | <0.0001* |
| Diabetes, n (%) | 20 (48.8) | 24 (47.1) | 0.8695 |
| Congestive heart failure, n (%) | 16 (39.0) | 33 (64.7) | 0.0141* |

* *p*<0.05

WMC, white matter changes; A-fib, atrial fibrillation

Patients with deep-WMC had a significantly greater incidence of hypertension and congestive heart failure, were more likely to be women, and were significantly older in comparison to those without deep-WMC.
