# Supplementary material for: The association between white matter changes and development of malignant middle cerebral artery infarction: A case–control study
Source: Medicine (Baltimore). 2021 Apr 30;100(17):e25751. doi: 10.1097/MD.0000000000025751 (PMC8084049; doi:10.1097/MD.0000000000025751)
Supplement: Supplemental Digital Content [file medi-100-e25751-s005.doc]

Table S5. Demographic data of patients with and without severe Deep-WMC

|  | Non-Severe deep-WMC  (n=60) | Severe  Deep-WMC  (n=32) | *p*-value |
| --- | --- | --- | --- |
| Sex (male), n (%) | 38 (63.3) | 10 (31.3) | 0.0033* |
| Age, years, mean (±SD) | 68.5 (12.4) | 76.2 (10.6) | 0.0039* |
| A-fib, n (%) | 23 (38.3) | 18 (56.3) | 0.0996 |
| Hypertension, n (%) | 36 (60.0) | 31 (96.9) | 0.0002* |
| Diabetes, n (%) | 28 (46.7) | 16 (50.0) | 0.7605 |
| Congestive heart failure, n (%) | 26 (43.3) | 23 (71.9) | 0.0090* |

* *p*<0.05

WMC, white matter changes; A-fib, atrial fibrillation

Patients with severe deep-WMC had a significantly greater incidence of hypertension and congestive heart failure, were more likely to be women, and were significantly older in comparison to those without severe deep-WMC
